# Broadband absorption enhancement in plasmonic nanoshells-based ultrathin microcrystalline-Si solar cells

**DOI:** 10.1038/srep24539

**Published:** 2016-04-15

**Authors:** Waseem Raja, Angelo Bozzola, Pierfrancesco Zilio, Ermanno Miele, Simone Panaro, Hai Wang, Andrea Toma, Alessandro Alabastri, Francesco De Angelis, Remo Proietti Zaccaria

**Affiliations:** 1 Istituto Italiano di Tecnologia, via Morego 30, 16163 Genova, Italy; 2 Università degli Studi di Genova, Via Balbi 5, 16126 Genova, Italy; 3 Rice University, Physics and Astronomy Department, Brockman Hall6100, Main MS-61, Houston, TX77005, USA; 4 Rice University, Laboratory for Nanophotonics, Smalley-Curl Institute, Houston, TX 77005, USA; 5 Ningbo Institute of Materials Technology & Engineering, Chinese Academy of Sciences, Ningbo 315201, PR China

## Abstract

With the objective to conceive a plasmonic solar cell with enhanced photocurrent, we investigate the role of plasmonic nanoshells, embedded within a ultrathin microcrystalline silicon solar cell, in enhancing broadband light trapping capability of the cell and, at the same time, to reduce the parasitic loss. The thickness of the considered microcrystalline silicon (μc-Si) layer is only ~1/6 of conventional μc-Si based solar cells while the plasmonic nanoshells are formed by a combination of silica and gold, respectively core and shell. We analyze the cell optical response by varying both the geometrical and optical parameters of the overall device. In particular, the nanoshells core radius and metal thickness, the periodicity, the incident angle of the solar radiation and its wavelength are varied in the widest meaningful ranges. We further explain the reason for the absorption enhancement by calculating the electric field distribution associated to resonances of the device. We argue that both Fabry-Pérot-like and localized plasmon modes play an important role in this regard.

One of the great challenges in photovoltaics is the reduction of cost over watt with respect to fossil-fuel technologies. The cost/watt ratio of generated electricity through photovoltaic devices is at least 1.5 times higher than the electricity generated from fossil fuels^1^. One of the most important factors influencing the cost/watt ratio is the active material (mostly crystalline silicon, c-Si). For the case of c-Si solar modules, 30–40% of cost/watt is due to the silicon substrate^2^,^3^.

An efficient and reliable approach for reducing the cost/watt ratio is based on thin film solar cell technologies^4^,^5^, where amorphous silicon (a-Si:H), microcrystalline silicon (μc-Si), cadmium telluride (CdTe) and copper indium gallium selenide (CIGS) can be used as active materials. In this work, we focus on thin film μc-Si solar cells^6^. Similarly to the crystalline Si, also μc-Si is an indirect bandgap semiconductor, with low optical absorption for wavelengths between 600 and 800 nm. In Fig. 1(a) the absorption spectrum of a ultrathin (300 nm-thick) μc-Si layer for a normal incident light is compared with the AM1.5G solar spectrum^7^. The plot clearly shows that the light is poorly absorbed by the microcrystalline silicon layer between 600 nm and 1100 nm, approaching zero absorption above 800 nm. This result can be explained by the optical properties of μc-Si as in Fig. 1(b). The absorption of light is the first step toward highly efficient solar cells, it is then necessary to adopt light-trapping to increase the active absorption especially when ultrathin μc-Si solar cells (~1/6 of the thickness of conventional μc-Si based solar cells^8^) are considered.

During the last decades, several light trapping architectures have been proposed, ranging from arrays of pyramids, to photonic crystals^9^ or plasmonic structures. In particular, plasmonics exploits the capability of micro/nano metallic objects of concentrating light at their effective surface.

To date three kinds of arrangements have been proposed for photovoltaic applications through plasmonic nanostructures: i) metallic gratings at both the front and back contacts of the solar cell. Both waveguide and plasmonic modes (localized and propagating) can be excited to enhance the absorption in the active material^10^; ii) metallic nanoparticles are placed on the top of the solar cell. Their role is to scatter the incident light preferentially into the microcrystalline silicon by exploiting its high refractive index. The result is the increase of the optical thickness of the active region which allows for the semiconductor substrate to absorb higher amount of electromagnetic radiation^11^,^12^; iii) metallic nanoparticles are embedded inside the semiconductor layer. They will act as nano-antennas, namely the plasmonic near-field enhancement of the electric field causes an increase of the effective absorption rate inside the semiconductor^13^.

An important issue emerging when metallic nanostructures are considered for PV applications is the photocurrent loss due to parasitic absorption in the metal^14^,^15^. Recently, Brown *et al.*^16^ proposed to cover metallic nanoparticles with thin dielectric layers in order to reduce the effects of parasitic absorption and recombination in case of dye sensitized solar cells. However, too thick dielectric layers would limit the local field enhancement inside the active layer, hence rendering the overall cell less effective. In a similar manner Paz-Soldan *et al.*^15^ successfully demonstrated the use of metallic spherical nanoshells in colloidal quantum dots thin film solar cells while Guilatt *et al.*^17^ illustrated the use of tubular metallic nanoshells with different geometries, embedded in 50 nm of Si, through 2-D numerical simulations. Recently, P. Cheng *et al.*^18^,^19^ theoretically showed that placing spherical nanoshells at the top of a silicon thin film layer, light absorption can be enhanced through the excitation of localized surface plasmons. Moreover, W. Zhang *et al.*^20^ demonstrated the use of embedded metallic nanoshells in perovskite thin film solar cells to achieve a reduced exciton binding energy, in turn free charges enhanced generation. All the mentioned works demonstrate how metallic nanoshells can indeed represent a very important tool for enhancing the performance of solar cells. However, there is yet the need of understanding which are the optimal conditions for maximizing the broadband absorption of the active material when the overall parameters of the cell are considered, particularly for the case ultra-thin microcrystalline solar cell. In fact, it is expected that not only the shape of the nanoshells can influence the absorption response of the active material, but similar effects can be attributed also to parameters such as periodicity, dimensions, incident angle and materials optical properties.

In the present work we designed a three dimensional model of plasmonic ultra-thin film solar cell by placing a periodic array^21^,^22^,^23^ of spherical metallic nanoshells inside a thin layer of μc-Si active material. The idea is to exploit their near-field concentration capability^24^,^25^ and, at the same time, to strongly suppress parasitic losses. The goal is the enhancement of the optical absorption in a wide spectral range (400–1100 nm) under AM1.5G solar radiation. In particular, by implementing the Finite Element Method (COMSOL) technique, we discuss the optical properties of metallic nanoshells and describe their permittivity as a function of the metallic shell thickness. Furthermore, the optimization study on metallic nanoshells array (size and period) to achieve the maximum optical enhancement was also performed. Finally, regarding an important aspect often neglected, we have gone beyond normal incidence assumption by investigating the role of the angle of incidence on the overall optical absorption enhancement. In fact, both TE and TM polarizations were taken into account to estimate the effect of the Sun un-polarized incident light on the proposed structure.

The manuscript is structured as follows. In the Results section, we introduce the structure under investigation and the simulation method followed by the description of the optical properties of metallic nanoshells. In this regard, we employ a modified metal permittivity capable of taking into account the scattering effects from the surface nanoshell. Afterwards, in the Discussion section, we proceed by optimizing the plasmonic nanoshells array in order to maximize the absorption of sunlight: special attention is paid to the effect of size, periodicity, incidence angle and polarization. Finally, the conclusions and future developments are provided.

## Results

### Proposed structure and simulation method

In our proposed architecture of thin film solar cells, the active region is formed by 300 nm of microcrystalline silicon sandwiched between 100 nm of aluminium, working as back electrode, and 1 nm thick multilayer graphene, having the function of transparent top conductive layer^26^. The choice of a graphene based top layer is motivated by the requirement of overcoming some of the issues related to the commonly used indium titanium oxide (ITO): increased cost of indium due to its scarcity^27^ and its sensitivity towards acidic and basic environment^28^. Furthermore, graphene possess excellent optical and electrical properties, particularly it provides higher transmission over a wider wavelength range with respect to ITO^29^. Finally, it also exhibits stronger mechanical properties, to the extent that it has been proposed as candidate to replace ITO and other transparent conductors^30^. Spherical shaped gold nanoshells particles are then embedded at the centre of the active region. Their pattern follows a periodic square array. The perspective view of the proposed architecture is shown in Fig. 2(a,b). The geometry of each nano-shell comprises of a dielectric core (SiO_2_) of radius R_1_ coated with gold metal (Au) shell of radius R_2_, as shown in Fig. 2(c). The period of nanoshells array is denoted with P.

Importantly, the realization of the proposed planar structure is readily possible. A feasible and comprehensible fabrication process to embed the nanoshells in the desired 2-D geometrical arrangement would contain the following steps^31^,^32^,^33^: firstly, micro-crystalline Si layer (300 nm) is deposited onto aluminium collector by low temperature physical vapour deposition (PVD), by Chemical Vapour Deposition (CVD) or by Plasma Enhanced Chemical Vapour Deposition (PECVD)^34^,^35^. Afterwards, the desired geometrical arrangement is created by lithography and gold nanoshells are placed by controlled evaporation and/or three-phase contact line motion. A detailed description of this step can be found in refs. 33,35. Later on, fabrication residuals and undesired organic material (e.g. nanoshell ligands) are removed by RCA cleaning. Finally, poly-crystalline Si is deposited by CVD^35^ until the desired thickness is reached.

The optical constants, i.e. the wavelength dependent refractive index n + ik of microcrystalline silicon material is taken from O. Isabella *et al.*^36^ while Al is taken from Palik^37^ and the graphene optical properties are defined according to refs 38,39. Similarly, the Au shell optical constants are analytically calculated by implementing considerations on the shell thickness into the standard Drude-Lorentz formalism^40^. The overall system is illuminated from the top with a linearly polarized plane wave impinging with an angle θ with respect to the surface normal. In particular, the wavelength ranges from 400 nm to 1100 nm (AM1.5G illumination). Simulations were performed for an infinitely extended square array of nanoshells by applying periodic boundary conditions along x and y directions, as defined in Fig. 2(c). Perfectly matched layer (PML) boundary conditions were instead used both at the top and bottom boundaries. Once illuminated, the gold nanoshells are expected to leverage strong light absorption through the excitation of various modes in the μc-Si active material.

**Figure 1:**
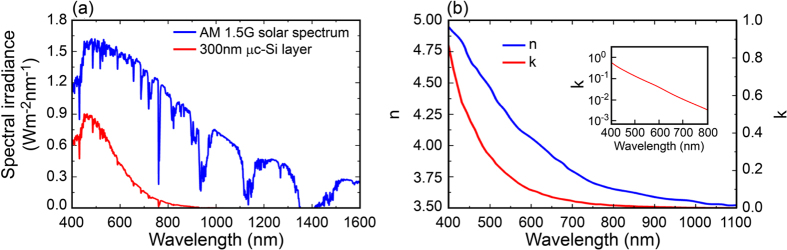
(**a**) AM1.5G global solar radiation (blue line) compared with the solar radiation absorbed by 300 nm of μc-Si (red line). A strong absorption decay is observed by increasing the wavelength. This behaviour well matches with the optical properties of μc-Si as shown in (**b**). In particular, both the real (n) and imaginary part (k) of μc-Si refractive index are shown. The inset highlights the small value of k, with special focus on the spectral range providing the highest spectral irradiance. In (**a**) light was assumed to impinge normally to the μc-Si surface.

**Figure 2:**
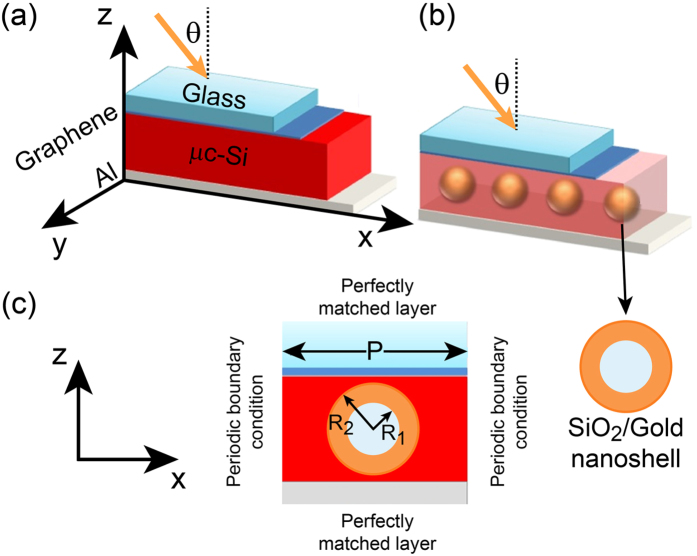
Scheme of the proposed thin film solar cell architecture. (**a**) Bare (no gold nanoshells) glass/graphene/μc-Si/Al as reference structure; (**b**) dispersed SiO_2_/Au nanoshells arrays in glass/graphene/μc-Si/Al configuration. (**c**) 2-D cross section of the architecture. As impinging light is assumed AM1.5G spectrum.

To calculate the absorption efficiency A_*eff*_ (*λ*) of the μc-Si active layer, namely its capability of capturing light, we integrated the divergence of the Poynting vector 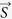 on the μc-Si volume and normalized it with respect to the input power flow *P*_*in*_^41^,^42^:


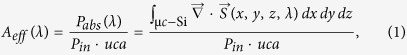


where *P*_*abs*_(*λ*) is the absorbed power in the μc-Si layer before normalization, 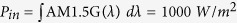 is the total incident power being AM1.5G the global solar irradiance striking on Earth surface (see Fig. 1(a)) and *uca* is the unit cell area. The absorption efficiency was calculated for two configurations, that are cells with (Fig. 2(b)) and without (Fig. 2(a)) embedded nanoshells. In fact, the outcomes were utilized for estimating the photocurrent enhancement J_*enh*_ of the cell, defined as^43^,^44^:


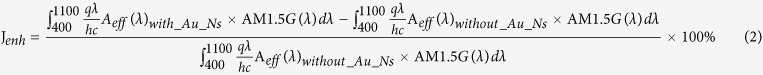


The wavelength range from 400 nm to 1100 nm was chosen based on the optical response of μc-Si as in Fig. 1(a).

### Surface Plasmon and optical properties of metallic nanoshells

Owing to the advancement of fabrication and characterization techniques, new types of composite nanoparticles have been designed with the characteristic of exhibiting strong optical resonances. Metallic nanoshells are the prime example of these kinds of composite nanoparticles^45^. In terms of photovoltaic applications, nanoshells can be utilized to enhance the light absorption into the semiconductor layer of thin film solar cells. Since nanoshells exhibit controlled optical resonances, they can be utilized as a promising structures to exploit plasmonic resonances for photovoltaic applications with a strongly suppressed parasitic loss.

**Figure 3:**
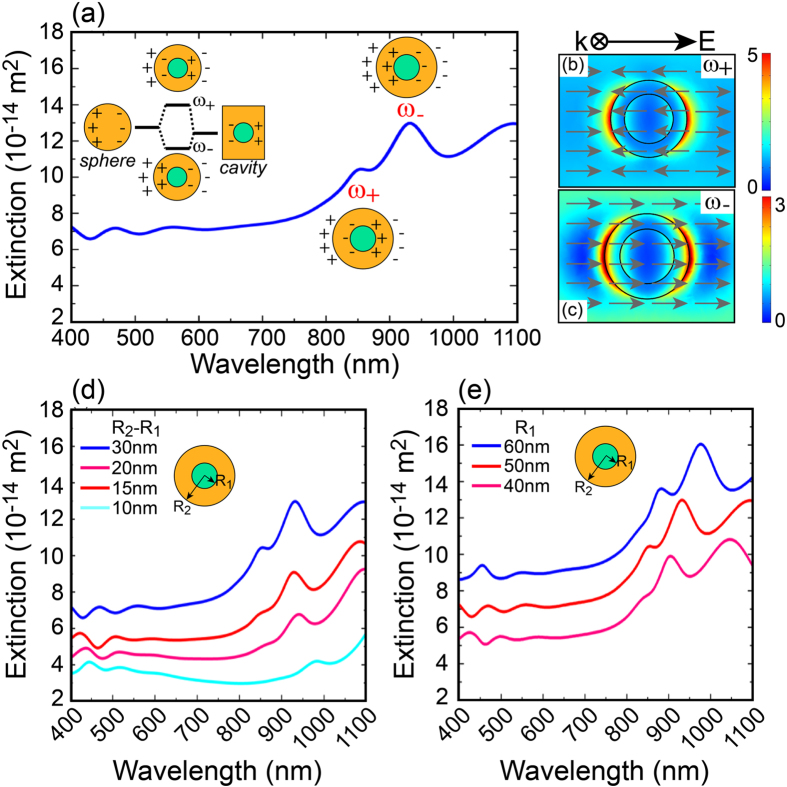
(**a**) Calculated extinction spectrum of a single spherical nanoshell with core radius R_1_ = 50 nm and Au thickness R_2_ − R_1_ = 30 nm embedded in μc-Si medium. Two plasmon peaks are observed at longer and shorter wavelengths, corresponding to symmetric (ω_−_) and anti-symmetric (ω_+_) modes, respectively. Inset: hybridization model for surface plasmon resonance in a nanoshell. The sphere and cavity modes interact with each other forming symmetric (ω_−_) and anti-symmetric (ω_+_) plasmon resonances. The plus/minus symbols in the figure describe the charge distribution. Normalized electric field norm (|E|/|E|_in_) for (**b**) symmetric mode (*λ* = 930 nm) and (**c**) anti-symmetric mode (*λ* = 850 nm). (**d**) Extinction spectra at different shell thickness with 50 nm radius silica core. (**e**) Extinction spectra calculated at varying core radius with shell thickness fixed at R_2_ − R_1_ = 30 nm.

The plasmonic resonances excited in the nanoshells can be explained by the plasmon hybridization model^46^, describing the hybridization of plasmonic modes supported at the interfaces between dielectric-core/metallic-shell and metallic-shell/surrounding medium. When the thickness of the metallic shell is thin enough, the two modes can interact with each other resulting in the splitting of their energy level into symmetric or low energy (bonding, ω_−_) mode and anti-symmetric or high energy (anti-bonding, ω_+_) mode, as shown in the scheme of Fig. 3(a). Indeed, the strength of the interaction between these modes is controlled by the thickness of the metal shell layer. If the thickness of the metallic shell is less than the mean free path of the conduction electrons (~30 nm for gold in the visible range), the electrons will tend to scatter with the metal surface. This phenomenon imposes a modification of the permittivity of the metallic shell^47^,^48^:


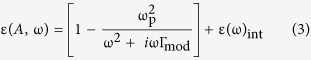


where


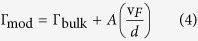


and v_*F*_ is the Fermi velocity (1.4 × 10^6^ ms^−1^)^48^, *d* is the shell thickness, *A* is a parameter related to the angular nature of electron scattering in case of metallic nanoshells (usually in the order of unity) and Γ_bulk_ is the bulk collision frequency. In Eq. (3) the first contribution is the Drude free electron term, where ω_p_ is the plasma frequency. The second term accounts for interband transitions of electrons which can be described by the Lorentz model^40^:


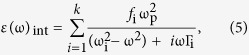


where *k* is the total number of oscillators each with frequency ω_i_, strength *f*_i_ and lifetime Γ_i_.

The first step towards the 3-dimensional modelling of a complete plasmonic solar cell is to calculate the optical response of a single silica/gold spherical nanoshell. The value of the permittivity for the SiO_2_ core is ε_c_ = 2.25 while the Au shell is calculated according to Eq. (3). Furthermore, μc-Si was chosen as surrounding material. This simple system was illuminated with a linearly polarized plane wave of amplitude 1 V/m and perfectly matched layers (PML) were used to avoid any reflection from the boundaries into the computational domain. In Fig. 3(a) is shown the extinction spectrum from a silica/gold nanoshell of core size R_1_ = 50 nm and shell thickness R_2_ − R_1_ = 30 nm in the wavelength range from 400 nm to 1100 nm. Two peaks appear in the extinction spectrum which are associated to the localized plasmon resonances excited in nanoshells: symmetric at *λ* = 930 nm and anti-symmetric at *λ* = 850 nm. Figure 3(b,c) depicts the corresponding electric field distributions where both amplitude and field-lines are shown. Importantly, the latter confirms the symmetric and anti-symmetric nature of ω_−_ and ω_+_ modes, respectively. In Fig. 3(d) is illustrated the far field optical response of the nanoshell by changing its metallic thickness, while keeping the core size constant at R_1_ = 50 nm. The graph shows a shift of the plasmonic resonances towards longer wavelengths by decreasing the shell thickness. Similar effect is obtained by increasing the core size of the nanoshell while keeping a constant shell thickness (R_2_ − R_1_ = 30 nm), as shown in Fig. 3(e). These results suggest that nanoshells can be very suitable structures for efficient tuning of the optical response of a solar cell for a best harvesting of the solar radiation.

**Figure 4:**
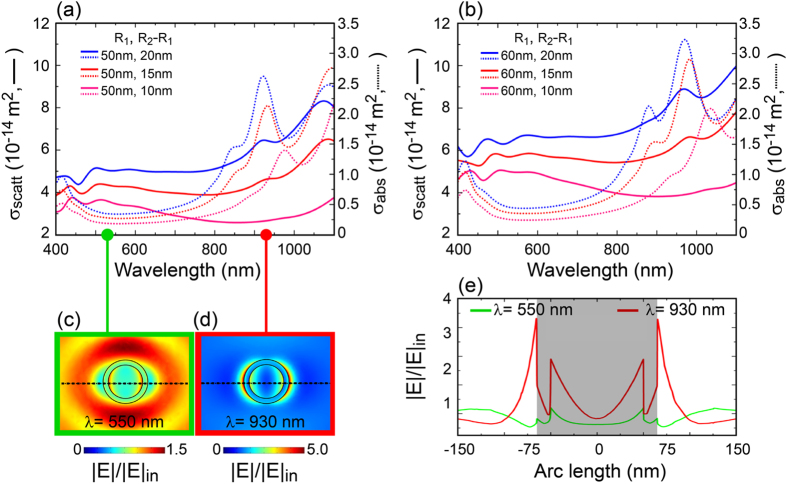
Calculated σ_scat_ (solid line) and σ_abs_ (dotted line) for isolated nanoshells with two different metallic core sizes (**a**) R_1_ = 50 nm, (**b**) R_1_ = 60 nm. For both cores various shell thickness (10 nm, 15 nm, 20 nm) are considered. Similarly, σ_abs_ (dotted line) is also reported. (**c,d**) represent the near field plots respectively calculated at *λ* = 550 nm and *λ* = 930 nm for a nanoshell with R_1_ = 50 nm and R_2_ − R_1_ = 20 nm. In (**e**) the normalized field profiles calculated along the dotted lines of (**c,d**). The shadow area represents the nanoshell. In all figures the chosen surrounding material is μc-Si.

Metallic nanoshells, similarly to any plasmonic structure, undergo parasitic absorption, namely the loss of photons inside the metal in form of heat. The tentative is then to reduce this detrimental effect in order to maximize the chance of absorption of photons in the active material. It is then important to keep into account both the absorption cross section (which should be minimized) and the scattering cross section (which should be maximized) of the nanoshell. Figure 4(a,b) illustrates both σ_scat_ (solid line) and σ_abs_ (dotted line) for a single nanoparticle by varying its core radius R_1_ and the metallic shell thickness R_2_ − R_1_ in a broad range of wavelengths when μc-Si is considered as surrounding material. The chosen range of wavelengths is from 400 nm to 1100 nm, having kept into account the band gap of the μc-Si (see Fig. 1(a)). In particular, the result shows that by increasing either the metal thickness or the core radius the absorption cross section σ_abs_ (i.e. parasitic loss) increases as well^49^,^50^. Finally, increasing the core or decreasing the shell thickness lead to a red shift. From these observations we can conclude that core dimensions and shell thickness are both accountable for increasing the scattering to absorption ratio and reducing the parasitic absorption. In particular, it is expected that a specific combination of core radius and shell thickness should provide the ideal condition where σ_scat_ is maximized and σ_abs_ is minimized. Noticeably, the figures demonstrate also that few nanometers change in the geometry of the nanoshell can imply strong difference in the overall optical response. Hence, from this result we can infer about the use of nanoshells for a fine tuning of the optical response of plasmonic solar cells.

After addressing the issue of parasitic absorption, an important aspect to be considered is the spatial distribution of the scattered field. In fact, the ideal situation is a field mainly concentrated in the active material and, at the same time, not too close to the electrodes in order to provide enough space/time for the occurring of the photon-exciton conversion. Under linearly polarized light source, the near-field pattern produced by a SiO_2_/gold nanoshell is given in Fig. 4(c,d). In particular, a nanoshell of core radius R_1_ = 50 nm and shell thickness R_2_ − R_1_ = 20 nm has been considered. In Fig. 4e is shown the corresponding normalized electric field calculated along the dotted lines of Fig. 4(c,d). These plots demonstrate that light tends to move away from the nanoshell into the active material at off resonance conditions (green line), well matching with the far-field graphs of Fig. 4(a). It is then matter of proper nanoshell engineering, in order to have a field enhancement extended as much as possible into the active material.

## Discussion

Various geometrical parameters can be tuned to optimize the overall performance of the introduced plasmonic solar cell. In particular, we have shown how the nanoshells size (both core and shell) can induce a shift in the plasmon resonances (Fig. 3). Furthermore, the cell depicted in Fig. 2 shows a regular pattern of nanoshells with inter-particle distance P. In this regard, we can expect that the mutual interaction among the nanoshells can also influence the optical characteristics of the cell. Finally, another parameter besides geometry of the nanoshell and periodicity will be considered: the incident angle of the solar radiation.

**Figure 5:**
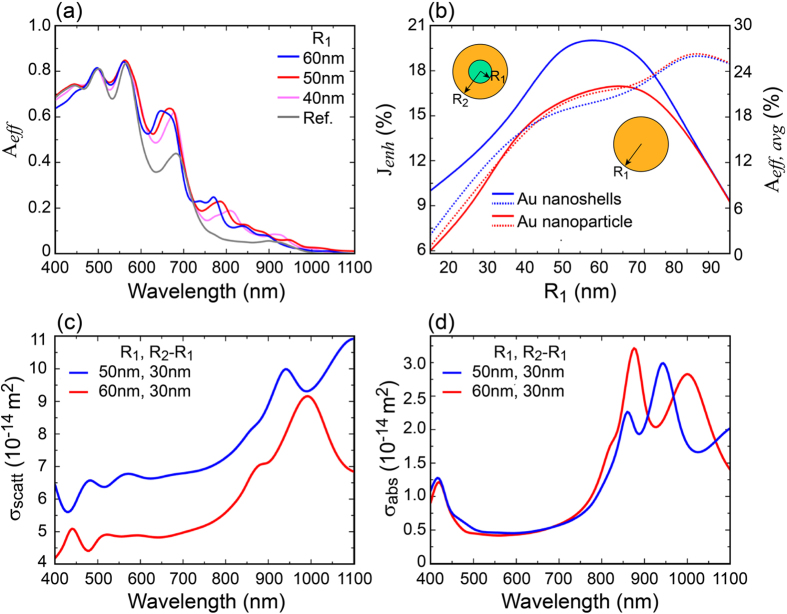
(**a**) Calculated absorption efficiency A_*eff*_ for 300 nm of μc-Si with embedded spherical silica/gold nanoshells computed for different core radius R_1_ and shell thickness equal to 30 nm The Ref. line describes the A_*eff*_ calculated for the configuration with no nanoshells (glass/graphene/μc-Si/Al, Fig. 2(a). (**b**) Calculated photocurrent enhancement when either nanoshells (blue-solid line) or spherical gold nanoparticle (red-solid line) are embedded into the μc-Si layer. Wavelength averaged absorption efficiency for both nanoshell (blue-dot line) and nanoparticle (red-dot line). For both figures (**a,b**) the period P is chosen equal to 300 nm while the shell thickness is kept constant at R_2_ − R_1_ = 30 nm. (**c,d**) are respectively the calculated σ_scat_ and σ_abs_ for an isolated nanoshell with core size R_1_ = 50 nm, shell thickness R_2_ − R_1_ = 30 nm and gold nanoparticle R_1_ = 60 nm.

The dependence of absorption efficiency A_*eff*_ (see Eq. (1)) on the core size R_1_ of spherical SiO_2_/gold nanoshells is investigated in Fig. 5(a). In particular, the core size is varied from 40 nm to 60 nm while the shell thickness is taken equal to 30 nm and the period P is kept constant at 300 nm with a square-like pattern. Figure 5(a) demonstrates the improvement in light collecting capability when the nanoshells are embedded in the solar cell. In order to compare the overall performance of a cell when using either metallic nanoshells or metallic nanoparticles, in Fig. 5(b) has been plotted the calculated photocurrent enhancement J_*enh*_ when nanoshells (blue-solid curve) or nanoparticles (red-solid curve) are considered. The results show a better performance of the nanoshells for all the considered geometries. In fact, a photocurrent efficiency around 20% upon embedding of nanoshells with core radius R_1_ = 50 nm can be observed, a result remarkably higher than the best performance reached by employing metallic nanoparticles. Interestingly, a photocurrent plateau is found between R_1_ = 45 nm and R_1_ = 55 nm which, in terms of cell fabrication, allows for a relaxed fabrication approach. Furthermore, Fig. 5(b) shows also the wavelength averaged A_*eff,avg*_ absorption efficiency within the nanoshell/nanoparticle defined as:


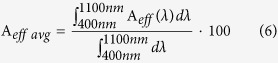


From the figure it can be noticed that around the region of maximum difference in current density between nanoshell and nanoparticle, the absorption within the nanoparticle overcomes the absorption inside the nanoshell. This result is even more interesting when the actual volumes of metal of the two configurations are compared: metal volume in the nanoshell is roughly 4 times higher than the metal volume in the nanoparticle which remarkably confirms the mitigation effect on parasitic absorption of nanoshells.

Figure 5(c,d) show respectively the scattering and absorption cross section from an isolated nanoshell of core radius R_1_ = 50 nm and shell thickness R_2_ − R_1_ = 30 nm (blue line) and from a gold nanoparticle of radius 60 nm (red line). These geometrical parameters correspond to the maxima in the current density plots of Fig. 5(b). The graphs clearly show that the nanoshell is characterized by higher scattering and lower absorption (i.e. parasitic) than the nanoparticle. In turn, this means that the nanoshell can provide higher chance of photon-exciton conversion associated to a lower heating, namely better photovoltaic performance than the nanoparticle.

We have seen that in the proposed design the parameter J_*enh*_ plays an important role in defining highly performing cells. In turn, this means specific optimum core radius and shell thickness. However, we have to recall that the present configuration considers 300 nm thick cells, therefore particular attention must be taken in avoiding proximity effects between the external layer of the nanoshell and the boundaries of the active material. In fact, this situation would lead to leakage of the radiation from the nanoshell into the outside medium which would have detrimental consequences on both the absorption efficiency A_*eff*_ and the photocurrent enhancement J_*enh*_.

**Figure 6:**
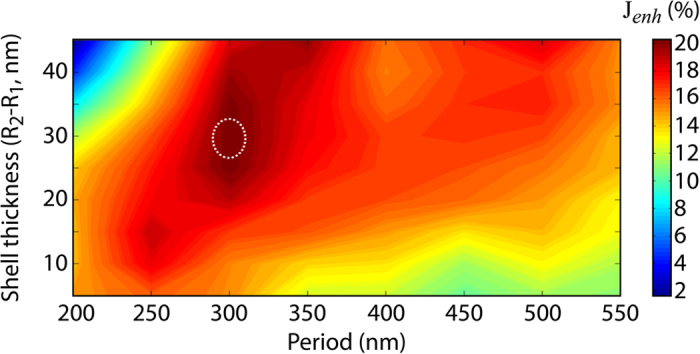
Photocurrent enhancement as function of shell thickness R_2_ − R_1_ and period. The spherical silica/gold nanoshell has core radius R_1_ = 50 nm. The dotted circle represents the conditions maximizing J_*enh*_.

In Fig. 5(b) is plotted the dependence of J_*enh*_ on the core radius R_1_ when the shell thickness is kept constant. Similarly, in Fig. 6 we investigate the influence of both the shell thickness and period P on the photocurrent enhancement when the core radius is kept equal to 50 nm. From the figure we can observe two important aspects: i) a monotone increase of J_*enh*_ with the shell thickness up to an ideal maximum. Afterwards, J_*enh*_ starts decreasing; ii) A clear dependence of J_*enh*_ on the periodicity. In particular, in the considered ranges of shell thickness and period P, the maximum (dotted circle in the figure) J_*enh*_ = 15.89 mA/cm^2^, corresponding to ≈20% enhancement with respect to a cell with no embedded nanoshells, is obtained for R_2_ − R_1_ ≈ 30 nm and P ≈ [280–340] nm. Interestingly, we can observe a smooth change of J_*enh*_ upon change either the shell thickness (as already seen in Fig. 5(b)) or the cell period. This aspect is quite important, in fact it ensures the possibility of fabricating the solar cell without the need of an absolute control of the geometrical parameters which, in turn, results in costs reduction.

We have found how the performance of thin film solar cells with embedded metallic nanoshells can be improved by tuning the geometrical parameters of the nanoshells together with the period of the associated array. From Fig. 6 we have identified an optimal shell thickness of 30 nm and a period between 280 nm and 340 nm when a core radius R_1_ = 50 nm is chosen. This optimized design of thin film solar cell with embedded spherical nanoshells, where graphene is used as front electrode, can improve the cell photocurrent of about 20% with respect to the reference structure of proposed design (no nanoshells).

**Figure 7:**
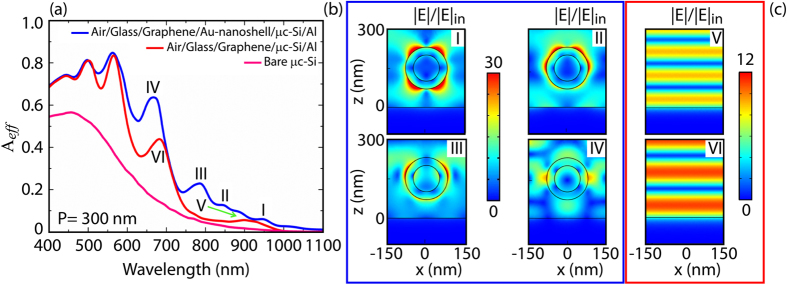
(**a**) Calculated absorption efficiencies for (i) optimized design formed by spherical silica/gold nanoshells of core radius R_1_ = 50 nm and shell thickness R_2_ − R_1_ = 30 nm embedded in a 300 nm thick μc-Si layer. Period equal to 300 nm (blue line); (ii) as above without nanoshells (red line); (iii) 300 nm thick μc-Si layer surrounded by air (pink line). Notice the broadband absorption characteristic promoted by the use of nanoshells. (**b**) Normalized magnitude of the electric field for the optimum structure under AM1.5G illumination with impinging light polarized along x and propagating along z direction. The wavelengths associated to the four peaks of the blue curve in (**a**) are considered: (I) *λ* = 950 nm; (II) *λ* = 850 nm; (III) *λ* = 790 nm; (IV) *λ* = 660 nm. (**c**) Near field representation of the Fabry-Perot modes shown by the red curve in (**a**). The modes are well confined between the Al and Graphene layers.

In Fig. 7(a) are shown the absorption efficiencies of the optimized design, the same optimized design without embedded nanoshells and a bare μc-Si substrate. Clearly, the use of metallic nanoshells improves the absorption efficiency all over the broad wavelength range from 400 nm to 1100 nm. By analysing the near field response in Fig. 7(b), corresponding to the peaks I–IV of Fig. 7(a), we can observe that the absorption improvement is mainly originating from the electric field enhancement associated to the optical resonances developed by the nanoshells. In general, the resonances of a cell can be categorized into two types: Fabry-Perot (FP) modes and localized plasmon (LP) modes. In particular, ideal FP modes describe light experiencing confinement due to the reflection from both Al/μc-Si and graphene/μc-Si interfaces, as shown in Fig. 7(c). In case of a LP mode the electric field is instead greatly confined in the region around the nanoshell. Importantly, these two modes are not each other orthogonal, meaning that they can coexist to form a more complicated electric field pattern.

The mode I, associated to *λ* = 950 nm, can be described by a quadrupole LP mode, namely the light absorption in the μc-Si is mainly occurring around the nanoshell. With the mode II (*λ* = 850 nm) the situation starts changing: a hexapolar LP mode can be noticed together with a FP mode, namely a larger volume of μc-Si participates to the absorption of light. Similar behaviour is observed also at smaller wavelengths, at peak III (*λ* = 970 nm) and peak IV (*λ* = 660 nm). Finally, the peaks V (*λ* = 930 nm) and VI (*λ* = 690 nm) well describe pure FP modes sustained by the nanoshell-less structure which, in case of configuration with embedded nanoshells, contribute to the formation of the modes I and II. Importantly, these results highlight the contribution of the nanoshells to the absorption efficiency especially in the spectral region where μc-Si demonstrates low absorption capabilities (above 600 nm).

**Figure 8:**
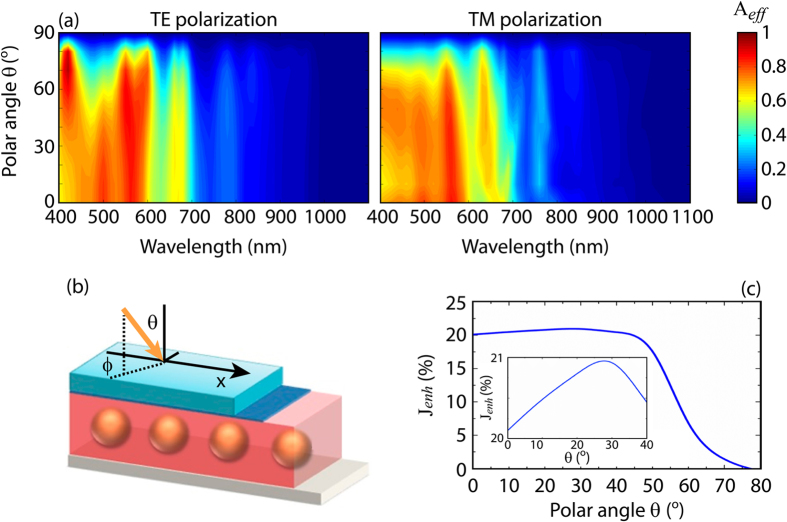
(**a**) Absorption efficiency vs polar angle/wavelength for a 300 nm μc-Si film solar cell with embedded spherical silica/gold nanoshells of core radius R_1_ = 50 nm and shell thickness R_2_ − R_1_ = 30 nm. Both TE (electric field // Y) and TM (electric field // X) polarizations are shown. (**b**) Schematic diagram of the solar cell, representing the polar and azimuthal angles. (**c**) Photocurrent enhancement J_*enh*_ with respect to the reference structures under AM1.5G illumination (inset: close view around 20° polar angle). In the calculation both TE and TM polarizations have been considered.

The performance of thin film solar cells with respect to the incident angle is a very important issue, even though many times neglected. Indeed, the normal impinging condition is, in a real system, occurring only for a very short lapse of time. In the present case we assume the azimuthal angle ϕ equal to zero while the polar angle θ is varied from 0° to 90° (see Fig. 8(c)). In Fig. 8(a,b) the absorption efficiency is shown in terms of *λ* and incident angle θ, when TE and TM polarizations are separately considered. In particular, it is shown the θ dependence of the absorption efficiency by varying the incident wavelength when the optimal structure is considered (R_1_ = 50 nm, R_2_ − R_1_ = 30 nm, P = 300 nm). It is noticed that above 60° the A_*eff*_ drops drastically in the all observed spectral range while two minima are found right above 800 nm and 1000 nm, as expected from the plots of Fig. 7(a).

In Fig. 8(d) the photocurrent versus the polar angle θ is instead shown. In this case the effect of TE and TM are averaged together thus providing a more realistic result resembling unpolarised light. The figure shows that the optimum value is obtained in the range between 20° and 30°, where a 21% enhancement is found. Importantly, the figure demonstrates the capability of the cell to efficiency collect light for a wide range of incident angles (0°–50°) while the actual drop in performance starts at 60°. This remarkable result reveals that the cell absorption efficiency is insensitive to the angle of incidence and makes it more suitable for real devices.

## Conclusion

We have proposed a design strategy for μc-Si thin film solar cells based on regularly embedded spherical silica/gold nanoshells to realize and optimize broadband absorption enhancement. The nanoshells were exploited in their capability of trapping light while maintaining low parasitic loss. A complete investigation of the role played by the geometrical parameters such as core diameter and metal thickness in the nanoshells and cell periodicity has been performed. The results show that by tuning these parameters an optimum photocurrent enhancement of about 21% can be reached yet preserving a robust optical response of the cell. Furthermore, mode analysis showed that a combination of Fabry-Pérot-like and plasmonic modes is responsible for the increased performance of the plasmonic cell. In particular, it was shown how the nanoshells play an important role especially at long wavelengths, where the active material shows minimum absorption. Finally, the cell optical performance was investigated upon change of the incoming angle. The results demonstrate that above 60° the absorption efficiency rapidly drops while it is roughly constant from normal incidence up to 50°, thus suggesting the feasibility of this approach especially in a real device.

In perspective, the concept of embedding nanoshells inside an active material is of course not limited to μc-Si, which was chosen as representative material. Indeed, other kinds of active materials can be chosen, such as a-Si:H, crystalline silicon, gallium arsenide, in fact this technique can be thought to be extended even to multi-junction solar cells^51^.

## Additional Information

**How to cite this article**: Raja, W. *et al.* Broadband absorption enhancement in plasmonic nanoshells-based ultrathin microcrystalline-Si solar cells. *Sci. Rep.* **6**, 24539; doi: 10.1038/srep24539 (2016).
